# A Novel Fluorescent Aptasensor for Arsenic(III) Detection Based on a Triple-Helix Molecular Switch

**DOI:** 10.3390/molecules28052341

**Published:** 2023-03-03

**Authors:** Min Yuan, Ye Yang, Nguyen Thi Quynh Chau, Qinqin Zhang, Xiuxiu Wu, Jiaye Chen, Zhiwei Wu, Heng Zhong, Yuanyuan Li, Fei Xu

**Affiliations:** Shanghai Engineering Research Center of Food Rapid Detection, University of Shanghai for Science and Technology, Shanghai 200093, China

**Keywords:** arsenic(III), fluorescent aptasensor, triple-helix molecular switch, rapid detection

## Abstract

**Highlights:**

- An ultra-sensitive fluorescence aptasensor employing a triple helix molecular switch was developed for the rapid detection of As(III).
- The proposed biosensor showed high sensitivity, and excellent selectivity in arsenic detection, with a detection limit of 69.95 nM and a wide linear range from 0.1 to 2.5 μM.
- The THMS sensing strategy developed herein can be extensively applied in food safety and environmental monitoring.

**Abstract:**

A novel aptamer-based fluorescent-sensing platform with a triple-helix molecular switch (THMS) was proposed as a switch for detecting the arsenic(III) ion. The triple helix structure was prepared by binding a signal transduction probe and arsenic aptamer. Additionally, the signal transduction probe labeled with fluorophore (FAM) and quencher (BHQ1) was employed as a signal indicator. The proposed aptasensor is rapid, simple and sensitive, with a limit of detection of 69.95 nM. The decrease in peak fluorescence intensity shows a linear dependence, with the concentration of As(III) in the range of 0.1 µM to 2.5 µM. The whole detection process takes 30 min. Moreover, the THMS-based aptasensor was also successfully used to detect As(III) in a real sample of Huangpu River water with good recoveries. The aptamer-based THMS also presents distinct advantages in stability and selectivity. The proposed strategy developed herein can be extensively applied in the field of food inspection.

## 1. Introduction

In the past few decades, arsenic pollution in groundwater has been a serious threat to global health [1]. More than 200 million people worldwide are exposed to arsenic contaminated groundwater where the concentration is far higher than the limit of 10 µg/L (133.47 nM) recommended by WHO [2]. The excessive intake and inhalation of As(III) can cause acute poisoning and long-term damage [3]. Therefore, the establishment of simple and convenient As(III) detection technology has important development prospects and practical significance.

Atomic absorption spectrometry (AAS) [4], atomic fluorescence spectrometry (AFS) [5], inductively coupled plasma mass spectrometry (ICP-MS) [6] and other detection methods relying on large instruments are the traditional methods for As(III) analysis. These analytical methods can reach a lower detection limit. However, the high cost of large-scale instruments limit its wide application in areas with serious As(III) pollution (mainly in developing countries). In addition, these methods are time-consuming with high technical requirements and a strict operating environment, so they cannot be popularized in a wider range [7]. To overcome these shortcomings, researchers are now turning to low-cost and fast sensing strategies [8].

Aptamer is a popular detection material at present, which can make up for some defects of traditional methods such as the high price and long reaction time [9]. Aptamers are single stranded DNA or RNA molecules that can bind to target molecules with high affinity and specificity by folding into different secondary and tertiary structures [10]. Compared with antibodies, aptamers have the advantages of small size, reversible denaturation, easy modification, slow degradation kinetics, non-toxicity and lack of immunogenicity [11]. These excellent biochemical properties make aptamers an ideal candidate recognition unit for developing biosensor systems for biological analysis [12]. In recent years, people have devoted themselves to the development of simple, cheap and selective As (III) detection sensors [13]. Among them, fluorescent aptamer detection has developed rapidly because of its high sensitivity, strong specificity, and simple and rapid reaction [14]. Based on the conformational change of aptamers during the binding process, various DNA molecular switches have been designed to construct convenient and fast aptamer fluorescence sensors [15]. Yu et al. [16] demonstrated an aptamer-based fluorescence anisotropy (FA) sensor for Cd^2+^ with a single tetramethylrhodamine (TMR)-labeled 15-mer Cd^2+^ binding aptamer. Compared with aptamers based on a double helix DNA molecular switch [17] and molecular beacon [18], the triple helix molecular switch [19,20] has the advantages of high sensitivity, good stability, retention of the original aptamer selectivity and affinity [21]. The triple helix molecular switch (THMS) consists of two DNA strands. One is a hairpin aptamer with a special sequence at both ends, and the other is a double labeled oligonucleotide signal transduction probe (STP) [22]. The triple helix structure means that when two chains form a double helix through a base complementary pairing, the third chain inserts into the big groove of the double helix structure through hydrogen bonding [23]. According to the type of the third chain inserted, if it is pyrimidine, the chain is inserted into the middle of the big groove of the double helix in a positive parallel way, that is, to form a pyrimidine purine pyrimidine type triple helix structure, which mainly has two forms of T-AT and C-GC [24]; if the third chain is purine, the chain is twisted into the original groove by reverse parallel force, forming two different ways of G-GC and A-AT. The two types of triple helix structures have attracted much attention due to their good stability, versatility and specificity.

Here, a fluorescence detection platform based on a triple helix molecular switch was designed for As(III) monitoring. THMS was used as sensing element, and a fluorescent group (FAM) and quenching group (BHQ1) were used as signal indicators. The triple helix structure would be destroyed as As(III) was added. Then, the fluorescence was quenched. Compared with those traditional methods, this work does not need complex instruments and is very simple to operate. The fluorescence sensor has high selectivity for As(III) in the presence of other competitive molecules. This design can detect a large number of samples within 30 min, which shows its efficient detecting performance. The real sample of river water was also tested to verify its practical applicability. The cost of one test was less than USD 1, which reflected the low cost of the design.

## 2. Results and Discussions

### 2.1. Experimental Principle

**Figure 1:**
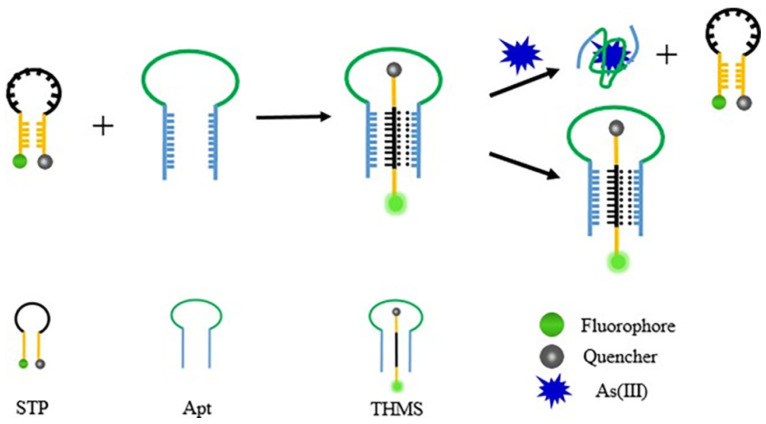
Schematic diagram of As(III) detection by triple helix molecular switch.

In the proposed THMS, the aptamer Apt contained 54 nucleotides in which the central segment can recognize and bind with As(III). Additionally, the terminal several bases of the Apt strand are designed to combine with STP to form a three helix molecular structure. As to the STP sequence, its heart segment is composed of continuous AG bases, and five bases at both ends form a hairpin structure through complementary pairing. The quenching agent (BHQ1) and fluorescent agent (FAM) are modified at 3′ and 5′ ends of STP as the fluorescent acceptor and donor, respectively. This means that the fluorescent end and quenching end of STP are close to each other through base complementation in a natural state. Additionally, the fluorescence quenched by nonradiative transferring energy from donor to acceptor. As shown in Figure 1, when the two chains are mixed they interact with each other through Watson–Crick and Hoogsteen to form a triple helix structure, which opens the complementary part of the STP chain and shows an open conformation. The fluorophore is far away from the quencher, causing fluorescence recovery. In the absence of As(III), the structure of the triple helix molecular switch remains stable. Otherwise, As(III) will combine with its aptamer to break the triple helix. The released STP will be folded into a closed hairpin structure by intramolecular hybridization, resulting in obvious fluorescence quenching. Therefore, As(III) can be quantitatively analyzed by monitoring the change of fluorescence intensity in the THMS system.

### 2.2. Feasibility of the Sensing Strategy

**Figure 2:**
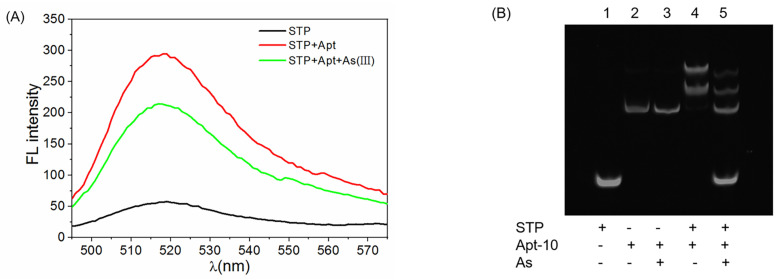
(**A**) Fluorescence spectra of STP, THMS in the absence and presence of 50 µM As(III). Excitation wavelength 480 nm. (**B**) Electrophoresis analysis. Lane 1: STP; Lane 2: Apt-10; Lane 3: Apt-10 and As(III); Lane 4: THMS; Lane 5: THMS and As(III).

The feasibility of the arsenic fluorescence biosensor employing the aptamer and the triple helix molecular switch was investigated. In Figure 2A, the fluorescence signals responses to the formation of THMS and the presence of As(III) were shown. For the STP with a hairpin structure, its fluorescence was very low due to the resonance transfer of energy between the fluorophore and quencher. After the addition of aptamer (Apt-8), the THMS structure was formed and its fluorescence signal increased to around 270 a.u at 518 nm. This is because the distance between the fluorophore and quencher was outspread with the unwinding of the hairpin structure of STP by binding with the terminal arms of aptamer. When As(III) ions were added, the fluorescence signal decreased obviously. It indicated that the aptamer was competitively dissociated from THMS, and some STP probes were released again. The feasibility of the strategy was also verified by a gel electrophoresis experiment, as shown in Figure 2B. The STP probe was released after adding As(III), which led to the fluorescence quenching. Therefore, the feasibility of the sensing method was validated.

### 2.3. Optimization of Experimental Conditions

**Figure 3:**
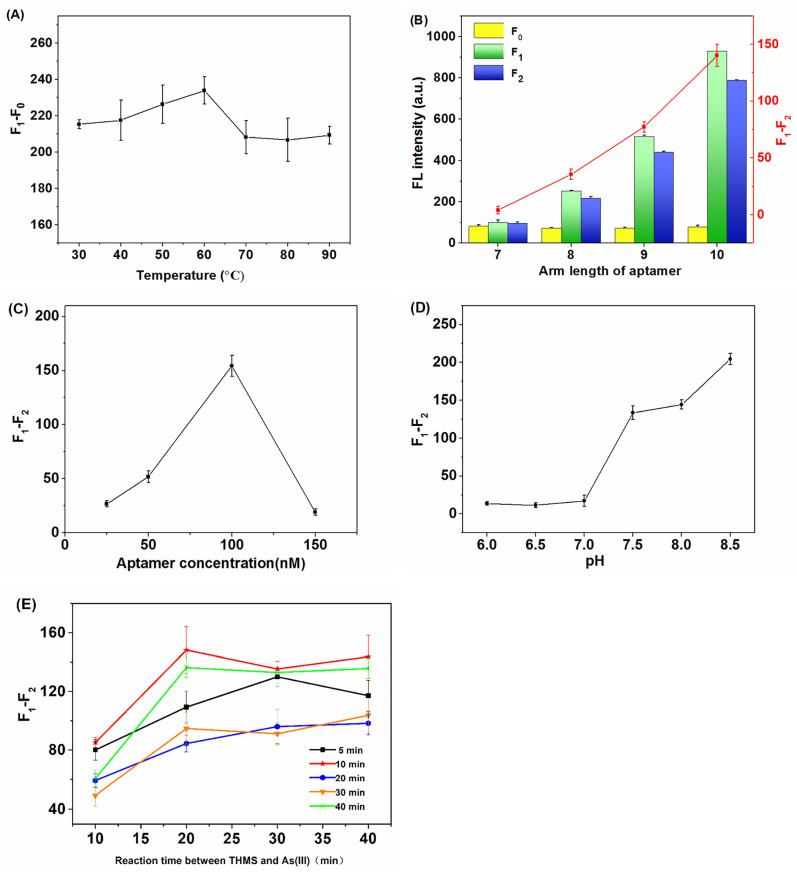
Optimization of the experimental factors on the fluorescence intensity of the sensing system. (**A**) STP unwinding temperature, (**B**) aptamer arm length, (**C**) aptamer concentration, (**D**) pH of the detected buffer, (**E**) reaction time. F_0_: fluorescence value of STP. F_1_: fluorescence value of the triple helix structure formed by STP and aptamer. F_2_: fluorescence value of THMS after As(III) addition.

In order to display a better arsenic detection performance, several parameters that may affect the sensing signal were optimized, including the STP unwinding temperature, the aptamer arm length, the aptamer concentration, and the pH of the buffer, etc. STP is a hairpin oligonucleotide chain composed of 20 bases, with five groups of consecutive AG bases to form a triple helix. In the natural state, STP is a hairpin structure because of the complementary pairing of terminal 5 base pairs. The quenching agent and fluorophore modified at both ends are close to each other, showing a fluorescence quenching phenomenon. In order to fully form the triple helix structure and maximize the recovery of the fluorescence signal, we firstly optimized the unwinding temperature range from 30 °C to 90 °C, with every 10 °C as a gradient. A total of 20 µL of 1 µM STP was placed at the set temperature for 10 min. Then, 40 µL of 1 µM aptamer Apt-8 and 340 µL of 20 mM PBS buffer solution were added and reacted for 10 min at 25 °C. The fluorescence value of STP and the triple helix structure formed by STP binding with aptamer were measured and recorded as F_0_ and F_1_, respectively. As shown in Figure 3A, the unwinding temperature of STP significantly affected the formation of its triple helix structure. When the temperature increased from 30 °C to 60 °C, the difference between F_1_ and F_0_ increased. It indicated that the binding degree of STP to the aptamer increased, due to appropriate heating promoting the unwinding and reassembly between bases. However, when the temperature exceeded 60 °C, overheating disrupted the stable binding between STP and aptamer so that the difference value of F_1_ − F_0_ decreased. Furthermore, the melting temperature of STP was also around 60 °C, according to the formula Tm = 4 (G + C) + 2 (A + T). Therefore, 60 °C was selected as the best melting temperature of STP in the following experiments.

In the process of THMS formation, the arm length of the terminal end of the aptamer can affect the spatial distance between the quencher and the fluorophore. Therefore, several aptamers (Apt-7, Apt-8, Apt-9, Apt-10) with arms of seven to ten bases were designed and tested. The results showed that the fluorescence of the triple helix gradually increased with increasing arm length (Figure 3B). When 5 µM As(III) was added, an obviously fluorescence quenching was induced when the arm length exceeded 8. Additionally, the quenching response of Apt-10 was much stronger than that of Apt-9 and Apt-8. Thus, Apt-10 was selected as the optimal capture sequence.

Following the aptamer sequence, the aptamer concentration was optimized in the range of 25 to 150 nM while the concentration of STP was fixed at 50 nM. As shown in Figure 3C, the 100 nM aptamer displayed the best quenching response to 5 µM As(III). Although the STP and aptamer should theoretically combine in a ratio of 1:1, a double supply of aptamers was actually more conductive to the full formation of THMS. At the same time, it should be noted that excess aptamer may affect the subsequent detection of As(III), so the final concentration of 100 nM was selected as the optimal aptamer concentration.

The pH of the reacted environment can affect the stability of the Hoogsteen base pairing, which may also have a great impact on the formation of the triple helix [25]; therefore, PBS buffer solutions (20 mM, containing 140 mM NaCl and 100 mM MgCl_2_) with pH 6.0 to 8.5 were used to test the impact of pH in arsenic detection. As shown in Figure 3D, F_1_ increased with the increase of the pH value in the range of 6.0 to 8.5. It indicated that the triple helix molecular structure was more fully formed in an alkaline environment. When As(III) was added, the fluorescence response reached the maximum at pH 8.5. The results run counter to the literature, which indicated that a weak acid environment was more favorable for Hoogsteen base pairing [26]. The reason for this phenomenon may be that the FAM fluorophore labeled at the terminal of STP is more stable in an alkaline environment. Additionally, the binding between As(III) and the aptamer may be stronger in a neutral and weak alkaline environment. These two factors had a more decisive influence than the pH environment of the base pairing. However, the formation of a triple helix molecular structure can be disturbed when the pH exceeds 8.5. Therefore, the buffer solution with pH 8.5 was selected in the subsequent experiments.

As shown in Figure 3E, different lines represented the reaction time of the aptamer and STP at the stage of triple helix formation, and the horizontal coordinate represented the reaction time when As(III) was added. According to the optimization of the two stages of triple helix formation and As(III) recognition, it could be seen that the fluorescence signal increased most significantly when the reaction time was 10 min in the formation stage of the triple helix. After the addition of As(III), the optimal effect was achieved after 20 min. However, if the reaction time was prolonged, the fluorescence decline value decreased slightly. Therefore, 10 and 20 min were selected as the optimal time of the first stage and second stage to react, respectively. The whole process was completed within 30 min, which was conducive to rapid detection.

### 2.4. Detection Performance of the Biosensor

**Figure 4:**
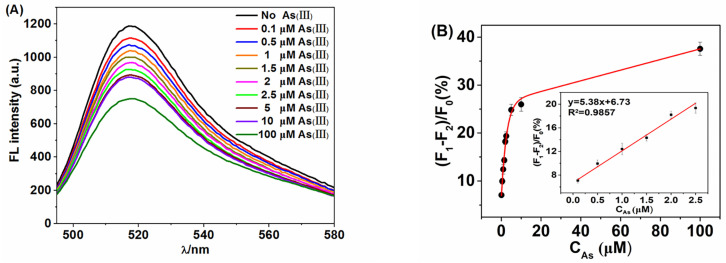
(**A**)Fluorescence spectra of the developed biosensor in the presence of different concentrations of As(III). (**B**) Linear relationship between fluorescence difference and concentration.

In order to evaluate the detection range and sensitivity of the method, various As(III) solutions in the concentration range of 0.1 to 100 µM were analyzed under the optimal conditions. As shown in Figure 4, the fluorescence intensity decreased with the increase in As(III) concentration. Additionally, the decrease in peak fluorescence intensity (F_1_ − F_2_) showed a linear dependence with the concentration of As(III) in the range of 0.1 µM to 2.5 µM. The equation was y = 5.38x + 6.73 (R^2^ = 0.9857). Additionally, the detection limit (LOD) was derived to 69.95 nM in accordance with 3σ. Compared with other analytical methods in Table 1, the proposed method shows a better LOD value which means that it is suitable for the assay of As(III).

### 2.5. Selectivity Analysis

**Figure 5:**
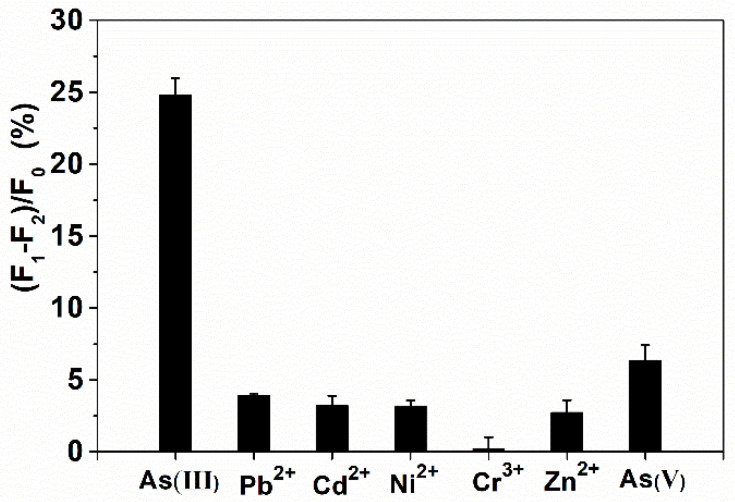
Selectivity analysis of the THMS biosensor towards As(III).

The selectivity of the biosensor was detected by comparing the fluorescence changes induced by 5 µM As(III) and 10 µM other interfering ions, including Pb^2+^, Cd^2+^, Ni^2+^, Cr^3+^, Zn^2+^ and As(V). The results showed that the other interfering ions did not cause significant interference to As(III) (Figure 5). Although As(V) has a slightly higher response than other metal ions, it is still much lower than As(III). Additionally, the developed THMS system displayed excellent selectivity to As(III). The raw data of Figure 5 can be viewed in Table S1.

### 2.6. Real Samples Analysis

The river water samples were spiked with As(III) to the concentrations of 0 µM, 0.2 µM, 1.2 µM and 2.2 µM, respectively. Additionally, the results detected by the developed strategy were shown in Table 2. The recovery ranged from 94% to 113%, with RSD (n = 5) lower than 8%. Hence, the established biosensor has wide practical value in the quick detection of As(III) in real samples.

## 3. Materials and Methods

### 3.1. Reagents and Materials

NaAsO_2_ was obtained from the China national standard network. NaH_2_PO_4_, Na_2_HPO_4_, C_4_H_11_NO_3_, Pb^2+^, Cd^2+^, N^i2+^, Cr^2+^, Zn^2+^ and As(V) standard solution were ordered from Sinopharm Chemical Reagent. All the other chemicals were of analytical grade.

All the oligonucleotides were synthesized by Sangon Biological Engineering Technology & Co. Ltd. (Shanghai, China) and the sequences are listed as follows.

STP: 5′-FAM-GAGGA GAGAG AGAGA TCCTC-BHQ1-3′

Apt-10: 5′-CTCTC TCTCT ACAGA ACAAC CAACG TCGCT CCGGG TACTT CTTCT CTCTC TCTC-3′

Apt-9: 5′-CTCTC TCTTA CAGAA CAACC AACGT CGCTC CGGGT ACTTC TTCCT CTCTC TC-3′

Apt-8: 5′-CTCTC TCTAC AGAAC AACCA ACGTC GCTCC GGGTA CTTCT TCTCT CTCTC-3′

Apt-7: 5′-CTCTC TCACA GAACA ACCAA CGTCG CTCCG GGTAC TTCTT CCTCT CTC-3′

The underlined parts are the aptamer arms for the formation of THMS. The identical 34 bases in the middle are the main identification sequences towards As(III). This aptamer was screened in a previous study [32] and optimized in this work.

The cleaning water used in the laboratory was self-made by the laboratory. The experimental operation water was Yibao purified water, which was purchased from the Shanghai Auchan supermarket.

### 3.2. Apparatus

The pH meter (S210-K) and the electronic balance (FA2204B) were purchased from Mettler Toledo. The constant temperature mixer (MSC-100) was bought from Hangzhou Aosheng Instrument Co., Ltd., Hangzhou, China. The PCR instrument (TC-XP-G) was ordered from Hangzhou Bori Technology Co., Ltd., Hangzhou, China. The fluorescence spectrophotometer was purchased from Shimadzu Experimental Equipment Co., Ltd., Shanghai, China.

### 3.3. Detection of As (III) and Characterization

The extended aptamer chain was annealed at 95 °C for 5 min and then slowly recovered to room temperature to keep its original conformation. The STP was placed in a water bath at 60 °C for 10 min. After cooling to room temperature, three groups of 20 µL of 1 µM STP were taken. The first group was added with 40 µL PBS buffer, and the other two groups were both added with 40 µL of 1 µM aptamer. The reaction was carried out in a constant temperature reactor at 25 °C for 10 min. Then, 340 µL PBS buffer was added to the first two groups, and 340 µL As(III) solution was added to the third group as an experimental group. All the three groups reacted for 20 min at room temperature, and were quickly scanned by fluorescence spectrophotometer (excitation wavelength 480 nm, emission wavelength 518 nm, slit 5 nm). The feasibility was examined by 12% polyacrylamide gel electrophoresis (PAGE) gel staining with SYBR Gold. In lane 4 and lane 5, the mole ratio of STP to Apt-10 was 1:1.

### 3.4. Sample Preparation

In order to further explore the potential application of the new biosensor in the actual sample, Huangpu River water was sampled for a recovery test. Before use, all the river water samples were filtered with a 0.22 mm microporous membrane. The river water samples were spiked with As(III) to different concentrations.

## 4. Conclusions

In summary, an aptamer-based sensing platform was developed for the fluorescent detection of As(III) by taking advantages of the triple-helix molecular switch (THMS), which consists of a target specific aptamer sequence flanked by two short-armed segments and a dual-labeled signal transduction probe (STP) in the stem portion. This aptamer-based THMS design ensures the affinity and specificity of aptamers to the target, and the stability and sensitivity of the THMS helps to measure the target in a short time, so this platform acquires a good performance of convenience, sensitivity and rapidity, exhibiting a detection limit as low as 69.95 nM and excellent selectivity toward As(III). The other metal ions, including the other forms of arsenic, such as As(V), have little interference to As(III). The established method has been successfully applied for the As(III) determination in a real sample. Furthermore, the universality of the approach can be achieved by virtue of altering the aptamer sequence. It is expected that this THMS sensing platform could be generalizable for the detection and control of other toxic chemicals in solution. However, a weak alkaline environment is more suitable for aptamer reaction, and extreme acidic or extreme alkaline environments may limit the application of the method. Thus, enhancing the stability and environmental compatibility of the aptasensor is one of our next research concerns.

## Figures and Tables

**Table 1:** Comparison of analytical responses of materials in As(III) detection with other reported analytical methods.

Material	Method	LOD (nM)	Reference
Apt-AgNPs	Colorimetric	80.08	[27]
SPCE/paper disc	Electrochemical	173.51	[28]
Probe (L)	Colorimetric	96.10	[29]
Ars-3 aptamer	Colorimetric	80.08	[30]
Zn(cur)O	Fluorescence detection	1334.72	[31]
Fluorescent Aptasensor	Fluorescence detection	69.95	This work

**Table 2:** Determination of As (III) in real samples.

Samples	Added (µM)	Found (µM)	Recovery (%)	RSD (%)
The water of the Huangpu River	0	-	-	-
	0.2	0.19 ± 0.016	95	7.9
	1.2	1.28 ± 0.080	107	6.7
	2.2	2.48 ± 0.165	113	7.5

## Data Availability

Not applicable.

## Supplementary Materials

The following supporting information can be downloaded at: https://www.mdpi.com/article/10.3390/molecules28052341/s1, Table S1: The raw data of Figure 5.
