# Supplementary material for: A Novel Fluorescent Aptasensor for Arsenic(III) Detection Based on a Triple-Helix Molecular Switch
Source: Molecules. 2023 Mar 3;28(5):2341. doi: 10.3390/molecules28052341 (PMC10005410; doi:10.3390/molecules28052341)
Supplement: Supplementary file 1 [file molecules-28-02341-s001.zip › molecules-2236334-supplementary.pdf]

Table S1 The raw data of Figure 5.

Ions	F_1-F_2	Error
As(III)	297.94	13.8
Pb^{2+}	46.787	1.86252
Cd^{2+}	38.288	8.4273
Ni^{2+}	38.0625	4.79206
Cr^{3+}	2.23767	9.95224
Zn^{2+}	32.848	10.14415
As(V)	75.884	13.13239
